# Molecular Characterization of *TP53* Variants in Exons 4-8 and p53 Immunoexpression in a Mexican Colorectal Cancer Cohort

**DOI:** 10.3390/cancers18111678

**Published:** 2026-05-22

**Authors:** Fernando Daniel García-Ayala, María de la Luz Ayala-Madrigal, Jorge Peregrina-Sandoval, José Miguel Moreno-Ortiz, Anahí González-Mercado, Ramón Antonio Franco-Topete, Jesús Alonso Valenzuela-Pérez, Nelly Margarita Macías-Gómez, Beatriz Armida Flores-López, Melva Gutiérrez-Angulo

**Affiliations:** 1 Instituto de Genética Humana “Dr. Enrique Corona Rivera”, Centro Universitario de Ciencias de la Salud, Universidad de Guadalajara, Guadalajara 44340, Jalisco, Mexico; fernando.garcia9652@alumnos.udg.mx (F.D.G.-A.); luz.ayala@academicos.udg.mx (M.d.l.L.A.-M.); miguel.moreno@academicos.udg.mx (J.M.M.-O.); anahi.gonzalez@academicos.udg.mx (A.G.-M.); 2 Programa de Doctorado en Genética Humana, Centro Universitario de Ciencias de la Salud, Universidad de Guadalajara, Guadalajara 44340, Jalisco, Mexico; jorge.peregrina@academicos.udg.mx; 3 Departamento de Biología Celular y Molecular, Centro Universitario de Ciencias Biológicas y Agropecuarias, Universidad de Guadalajara, Zapopan 44600, Jalisco, Mexico; 4 Departamento de Microbiología y Patología, Centro Universitario de Ciencias de la Salud, Universidad de Guadalajara, Guadalajara 44340, Jalisco, Mexico; ramon.ftopete@academicos.udg.mx; 5 Servicio de Colon y Recto, Hospital Civil “Dr. Juan I. Menchaca”, Guadalajara 44340, Jalisco, Mexico; dr_jvalenzuela@hotmail.com; 6 Laboratorio de Genética Humana, Centro Universitario del Sur, Universidad de Guadalajara, Ciudad Guzmán 49000, Jalisco, Mexico; nelly.macias@cusur.udg.mx; 7 Departamento de Ciclo de Vida, Facultad de Medicina, Universidad Autónoma de Guadalajara, Zapopan 45129, Jalisco, Mexico; beatriz.flores@edu.uag.mx; 8 Departamento de Ciencias de la Salud, Centro Universitario de los Altos, Universidad de Guadalajara, Tepatitlán de Morelos 47600, Jalisco, Mexico

**Keywords:** *TP53* gene, colorectal cancer, somatic variants, Mexican patients, DNA-binding domain, p53, immunohistochemistry

## Abstract

Background/Objectives: Colorectal cancer (CRC) represents a major public health problem in Mexico and is among the malignancies with the highest morbidity and mortality. Alterations in *TP53* are frequent molecular events in tumors with chromosomal instability; however, information on *TP53* variants in the Mexican population, particularly in exons 4-8, remains limited. Exons 4-8 comprise the main coding region of the p53 DNA-binding domain; therefore, this study aimed to identify *TP53* variants in these regions and evaluate p53 protein expression by immunohistochemistry in sporadic CRC. Methods: Tumor samples from 142 patients who underwent surgical resection without neoadjuvant treatment were analyzed. DNA was extracted from tumor tissue. *TP53* exons 4-8 were amplified by polymerase chain reaction (PCR), and variants were identified by Sanger sequencing. p53 immunohistochemistry was performed in 40 tumors and 36 adjacent tissues, and nuclear expression was assessed using the Immunoreactivity Score. Results: Forty-three heterozygous variants were identified in 106/142 patients, representing 75% of the cohort. Thirty-one patients carried oncogenic variants, mainly clustered within the DNA-binding domain and involving hotspot residues such as Arg175, Tyr220, Gly245, Arg248, Arg273, and Arg282. Nuclear p53 expression was observed in 9/40 tumors, whereas all adjacent tissues were negative. Conclusions: *TP53* alterations in exons 4-8 are frequent and heterogeneous in this Mexican cohort. Integrating mutational profiling with p53 immunohistochemistry provides complementary information for the biological interpretation of these tumors, including variants of translational interest.

## Simple Summary

Colorectal cancer is an important health problem in Mexico, but there is still limited information about the molecular changes involved in this disease in Mexican patients. One of the most relevant genes in colorectal cancer is *TP53*, which plays an important role in controlling cell growth and maintaining genomic stability. In this study, we analyzed specific regions of *TP53* in tumor samples from Mexican patients with sporadic colorectal cancer and evaluated the expression of its protein, p53, by immunohistochemistry. We found that *TP53* alterations were frequent and diverse, particularly in regions that are important for the normal function of p53. These findings provide local molecular evidence that may help improve the biological understanding of colorectal cancer in Mexican patients and support future research focused on tumor classification and precision medicine.

## 1. Introduction

Colorectal cancer (CRC) is a public health priority due to its high incidence and mortality. In Mexico, GLOBOCAN 2022 estimated 16,082 new CRC cases and 8283 deaths, ranking it as the third most diagnosed cancer and the leading cause of cancer death in the country [1].

Chromosomal instability (CIN) is the most frequent pathway in sporadic CRC, reported in ~70% of tumors and associated with a poorer prognosis [2]. *TP53* mutation is a recurrent event in this pathway [3,4]. *TP53* encodes p53, a nuclear phosphoprotein that functions as a transcription factor; its tumor suppressor activity depends on the regulation of post-translational modifications that modulate its stability, subcellular localization, and transcriptional activity, allowing it to regulate cell cycle arrest, DNA repair, apoptosis, and senescence programs [5].

In CRC, *TP53* is altered in approximately 50–60% of cases, and ~90% of variants are in exons 4-8, which encode the DNA-binding domain (DBD, residues: 100–288), as reported in the IARC database [6,7]. A proportion of p53 mutants exert dominant-negative effects over the wild-type allele or acquire gain-of-function (GoF) properties, contributing to pro-tumoral programs (proliferation, invasion, cellular plasticity) and a more aggressive phenotype [8].

This diversity of variants is also reflected in distinct p53 immunohistochemical (IHC) patterns in CRC, where diffuse overexpression (>80% of intensely positive nuclei) is mainly associated with missense variants in the DBD, a null pattern (complete absence of tumor staining with positive internal controls) correlates with inactivating variants (nonsense, frameshift, splice) and a cytoplasmic pattern may be linked to alterations in nuclear localization signals or the tetramerization domain [9].

Based on this, the present study aimed to analyze the spectrum of somatic *TP53* variants and p53 expression by IHC in CRC from Mexican patients, providing local evidence to support a more precise and biological stratification and future translational studies.

## 2. Materials and Methods

### 2.1. Patients and Tissue Samples

Primary tumor and adjacent non-tumor tissue samples were obtained from 142 patients with sporadic CRC from western Mexico who underwent surgical resection at the Hospital Civil de Guadalajara “Dr. Juan I. Menchaca” (Jalisco, Mexico). None of the patients received neoadjuvant chemotherapy or radiotherapy. Immediately after resection, a 25–50 mg tumor fragment was collected and stored at −80 °C. Two independent pathologists confirmed the diagnosis of colon or rectal adenocarcinoma in all cases. Written informed consent was obtained in accordance with the Declaration of Helsinki, and the protocol was approved by the local bioethics committee (CI-01417).

### 2.2. DNA Extraction and Quantification

Genomic DNA was extracted from frozen colorectal tumor tissue using the High Purity PCR Template Preparation Kit (Roche Diagnostics GmbH, Mannheim, Germany; product no. 11796828001), following the manufacturer’s protocol. DNA concentration and purity were assessed using a Thermo Scientific Multiskan SkyHigh microplate spectrophotometer (Thermo Fisher Scientific, Waltham, MA, USA; catalog no. A51119600DPC). DNA extracts were stored at −20 °C until use.

### 2.3. PCR Amplification, Sanger Sequencing, and Variant Interpretation

Exons 4-8 of *TP53* were amplified by polymerase chain reaction (PCR) using primers previously described by Liu and Bodmer [10], and the resulting amplicons were verified by electrophoresis on 2% agarose gels stained with SYBR Green I. Sanger sequencing was subsequently performed to identify variants within exons 4-8 and their adjacent exon-intron junctions. When required, sequencing was repeated in duplicate or performed using complementary primers to confirm the detected variants. Electropherograms were analyzed using Chromas v2.1.6 and Sage Sanger Trace Alignment. All variants were described according to Human Genome Variation Society (HGVS) nomenclature using the MANE Select transcript NM_000546.6 (updated 23 July 2023). Variant-level evidence, including population frequency, previous reports, in silico predictions, and clinical annotations, was compiled using free the VarSome platform (https://varsome.com/, accessed on 17 February 2026) [11] and OncoKB (https://www.oncokb.org/, accessed on 17 February 2026) [12,13]. Somatic variant oncogenicity was assessed according to the framework proposed by Horak et al. [14].

### 2.4. Determination of p53 by Immunohistochemistry

IHC analysis was performed to evaluate p53 protein expression in paraffin-embedded colorectal tumor tissues in a selected subgroup of 40 tumors from the 142 sequenced cases. This subgroup mainly included tumors harboring oncogenic or likely oncogenic *TP53* variants. Additionally, some samples harboring benign variants were included. For the analysis, 4 μm tissue sections were obtained using a microtome and mounted on positively charged slides. Samples were automatically processed using the IntelliPATH FLX Slide Stainer System (Biocare Medical, Pacheco, CA, USA) with the anti-p53 primary antibody (Biocare Medical, Pacheco, CA, USA; catalog number CME298AK). Immunoreaction was visualized using 3,3′-diaminobenzidine (DAB) as the chromogen, and sections were counterstained with hematoxylin. p53 expression was assessed by a pathologist using the Immunoreactivity Score (IRS), a semiquantitative method that integrates staining intensity and the proportion of positive cells. p53 positivity was defined as nuclear staining in ≥10% of tumor cells, and the IRS was calculated by multiplying staining intensity by the percentage of positive tumor cells. According to the final IRS, cases were classified as negative (0–1), weak (2–3), moderate (4–8), or strong (9–12). p53 immunoreactivity was assessed using criteria adapted from Fedchenko and Reifenrath [15].

## 3. Results

### 3.1. Clinicopathological Characteristics

A total of 142 patients with sporadic CRC were included in the study. The demographic and clinicopathological characteristics of the cohort are summarized in Table 1. The cohort was predominantly composed of patients aged ≥50 years, with a slight male predominance. Tumors were more frequently located in the colon, and most cases were moderately differentiated and diagnosed at advanced TNM stages.

### 3.2. TP53 Somatic Variants

**Figure 1:**
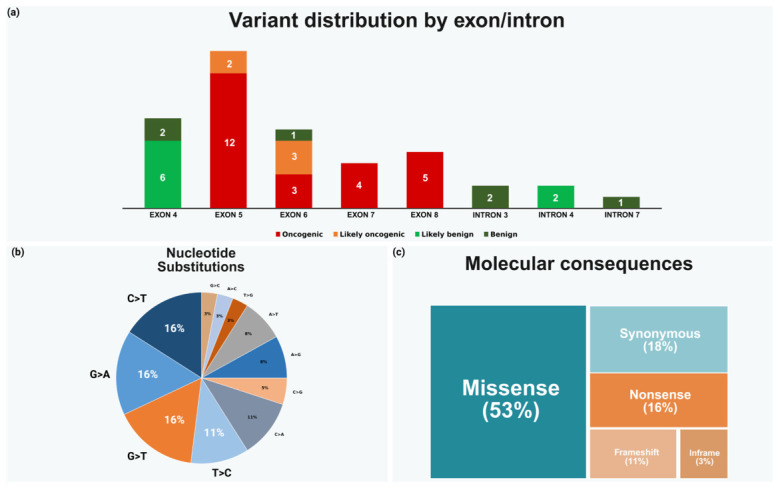
Overview of *TP53* variants identified in colorectal tumors. (**a**) Distribution of identified variants across exons and introns, classified according to oncogenicity. (**b**) Summary of the nucleotide substitution profile. (**c**) Molecular consequences of the detected variants. Created in BioRender. Gyc, L. (2026) https://BioRender.com/vua5ks8 (accessed on 22 April 2026).

**Figure 2:**
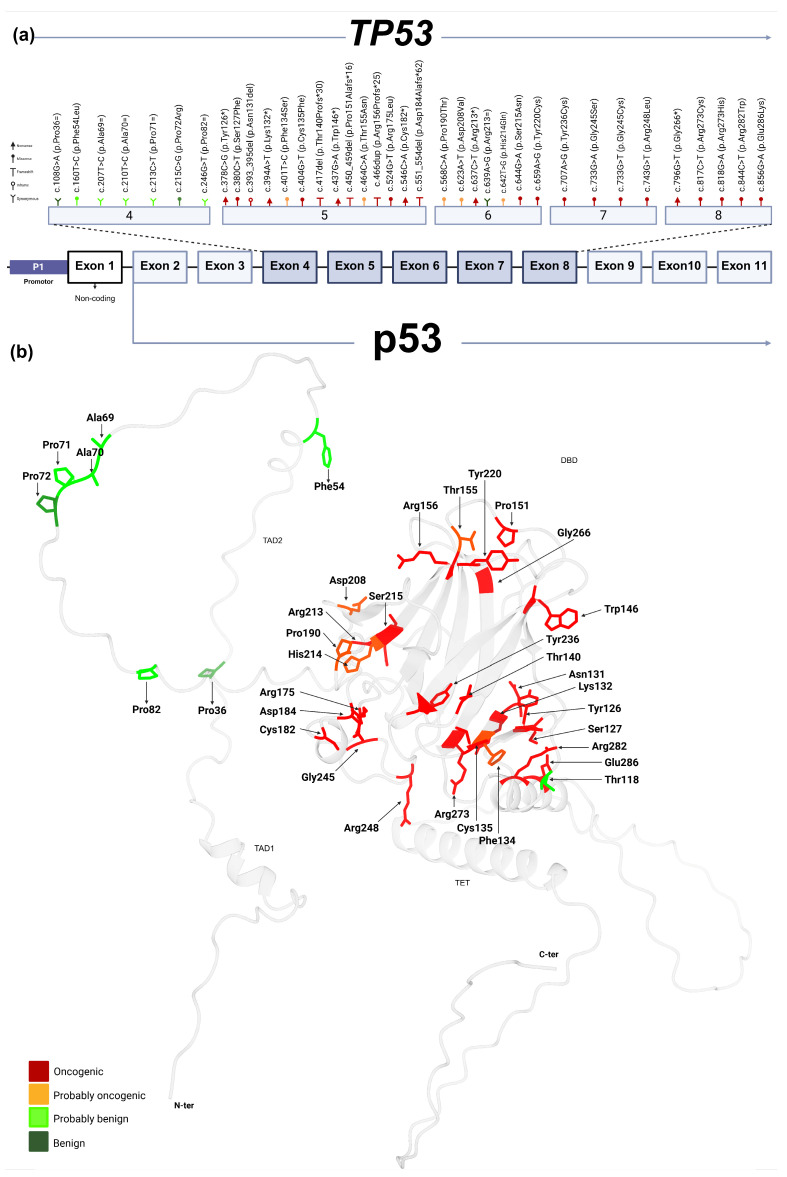
Genomic distribution and structural localization of *TP53* variants identified in colorectal cancer. (**a**) Genomic organization of *TP53* and localization of detected exonic variants. (**b**) Structural mapping of exonic variants onto the p53 protein model (AF-P04637-F1) [16], showing their localization across the main functional regions, including the transactivation domains, DNA-binding domain, tetramerization domain, and C-terminal region. Variant color coding indicates oncogenicity classification. Created in BioRender. Gyc, L. (2026) https://BioRender.com/74oexau (accessed on 22 April 2026).

Sequencing of *TP53* exons 4-8 in 142 colorectal cancer tumors identified 43 heterozygous variants, which were detected in 75% of patients (106/142), as summarized in Figure 1. At the protein level, arginine and proline were the residues most affected. A subset of patients (34/142, 25%) harbored more than one somatic variant. In these cases, the benign variant NM_000546.6:c.215C>G (p.Pro72Arg) co-occurred with other benign changes and with at least one additional oncogenic alteration located in the DBD. Thirty-one patients carried oncogenic *TP53* variants. Their distribution across the gene and protein is shown in Figure 2, highlighting clustering within the DBD and recurrence at canonical hotspot residues, including Arg175, Tyr220, Gly245, Arg248, Arg273, and Arg282. Detailed information for each identified variant is provided in Supplementary Table S1.

### 3.3. p53 Expression by Immunohistochemistry

**Figure 3:**
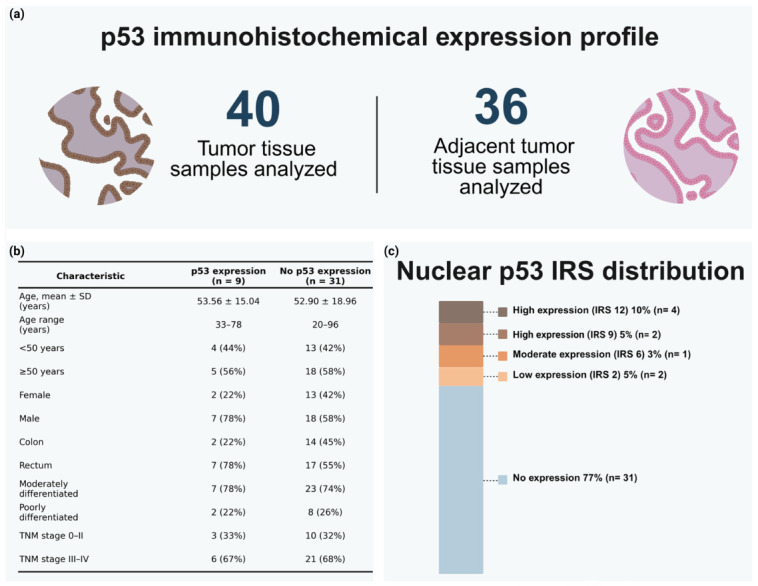
Profile of p53 immunohistochemical expression in tumor and adjacent tissues. (**a**) Overview of the tissue samples analyzed by immunohistochemistry, including 40 tumor tissues and 36 adjacent non-tumor tissues. (**b**) Clinicopathological characteristics according to p53 expression status in tumor tissues. (**c**) Distribution of nuclear p53 expression according to Immunoreactivity Score (IRS). Created in BioRender. Gyc, L. (2026) https://BioRender.com/7qfthxn (accessed on 22 April 2026).

**Figure 4:**
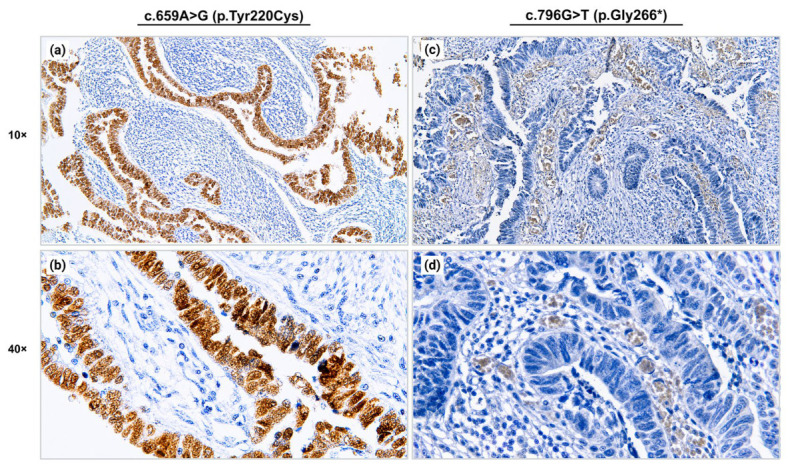
Representative p53 immunohistochemical expression patterns in colorectal cancer tumors. (**a**,**b**) Representative colorectal cancer tumor harboring the c.659A>G (p.Tyr220Cys) variant, showing strong and diffuse nuclear p53 immunoexpression at 10× and 40× magnification. (**c**,**d**) Representative colorectal cancer tumor harboring the c.796G>T (p.Gly266*) variant, showing loss of nuclear p53 immunoexpression at 10× and 40× magnification. Brown nuclear staining denotes p53-positive tumor cells, whereas blue hematoxylin counterstaining indicates nuclei without detectable p53 expression. Created in BioRender. Gyc, L. (2026) https://BioRender.com/oo8vjp5 (accessed on 22 April 2026).

The immunohistochemical profile of p53 in tumor and adjacent non-tumor tissues is summarized in Figure 3. Nuclear p53 expression was observed in 9 of 40 tumor samples (23%), whereas all adjacent non-tumor tissues were negative. Most tumors showed no expression, and the positive cases were distributed across low-, moderate-, and high-IRS categories. Representative immunohistochemical staining patterns observed in tumors harboring the *TP53* variants are shown in Figure 4. Detailed information on *TP53* variants according to p53 immunohistochemical expression status is provided in Supplementary Table S2.

## 4. Discussion

In the present study, we characterized *TP53* variants in a Mexican cohort of patients with CRC and described p53 immunohistochemical expression patterns in tumor and adjacent non-tumor tissues.

*TP53* variants were detected in 75% of cases (106/142), comprising 43 distinct variants. This frequency was comparable to that reported in recent CRC studies based on next-generation sequencing (NGS). Osakabe et al. [8] identified *TP53* variants in 72 of 92 Japanese patients, whereas Wu et al. [17] reported *TP53* variants in 230 of 294 Chinese patients, corresponding to frequencies of 78.3% and 78.2%, respectively. No statistically significant differences were observed between our cohort and those reported by Osakabe et al. [8] (*p* = 0.527) or Wu et al. (*p* = 0.404), indicating that the frequency of *TP53* alterations observed in Mexican patients is comparable to that recently described in other populations.

Regarding p53 immunohistochemical expression, our cohort showed nuclear positivity in 9 of 40 evaluated tumors, corresponding to 22.5%. This proportion was lower than that reported by Osakabe et al. [8] in Japanese CRC cases (41/92; 44.6%; *p* = 0.016) and by Wu et al. [17] in Chinese CRC patients (240/294; 81.6%; *p* < 0.001). These differences are most likely related to the fact that the IHC analysis in our study was performed in a selected, non-random subgroup of tumors enriched for oncogenic or likely oncogenic variants associated with loss of expression. It has been described that missense variants may promote stabilization and nuclear accumulation of mutant p53, whereas nonsense or frameshift variants may be associated with absent or low protein expression due to transcript degradation or the production of unstable truncated proteins [8,18]. Therefore, the IHC results are presented as descriptive observations and should not be interpreted as a representative estimate of p53 immunoexpression prevalence in the entire cohort.

*TP53* alterations have been widely studied in international cohorts; however, Mexican and Latin American populations remain underrepresented in cancer genomic studies. In Mexico, Luna-Pérez et al. [19] evaluated p53 overexpression by immunohistochemistry in patients with locally advanced rectal adenocarcinoma treated with induction chemoradiotherapy. In that study, p53 overexpression was observed in approximately 54% of cases, and its potential association with response to neoadjuvant treatment was analyzed, providing early evidence on p53 expression in rectal cancer in the Mexican population.

In Chile, Roa et al. [20] evaluated CRC samples by direct Sanger sequencing of *TP53* exons 5-9 and p53 immunohistochemistry. The authors reported point mutations in 60% of the analyzed carcinomas (21 of 35 cases) and indicated that p53 immunohistochemical detection showed a high predictive value for identifying genetic alterations. More recently, dos Santos et al. [21] performed mutational profiling using an NGS panel of 150 cancer-related genes in 91 Brazilian colorectal tumors. In that cohort, *TP53* was one of the most frequently altered driver genes, with mutations detected in 56.0% of tumors.

Collectively, these studies indicate that the available evidence in Mexican and Latin American patients with CRC remains limited. Therefore, our study contributes relevant local evidence by analyzing *TP53* variants in exons 4-8 together with p53 immunohistochemical expression in tumor tissue from Mexican patients with sporadic CRC.

Among the variants distributed across exons 4-8, a predominance of variants located in exon 5 was observed. This pattern is biologically consistent with the observation that most *TP53* missense mutations cluster within the central region of the gene, particularly in exons 5-8, which encode most of the DBD. The predominance of exon 5 in our cohort may be explained by the hypermutability of CpG dinucleotides. C>T transitions at CpG sites represent one of the most prevalent mutational classes in human cancer and are closely linked to 5-methylcytosine deamination, as well as to the high mutational susceptibility of CGN (CGA, CGT, CGC, CGG) codons encoding arginine. The hypermutability of CGN codons promotes both the recurrence of missense mutations in driver genes and the conversion of CGA to TGA, generating premature stop codons in tumor suppressor genes [22,23]. Although the design of our study does not allow direct attribution of the origin of each substitution, the predominance of C>T and G>A changes observed in our cohort is consistent with mutational mechanisms related to the hypermutability of CpG dinucleotides.

Hotspot mutations located within the DBD have well-established structural and functional consequences [24]. In our cohort, the conformational variants p.Arg175Leu and p.Gly245Ser were identified in tumors with negative p53 immunoexpression. These alterations cause DBD unfolding and may reduce the functional affinity for Zn^2+^, thereby promoting misfolding and loss of p53 transcriptional activity [24,25]. The absence of immunohistochemical staining in both cases could be consistent with low protein abundance due to structural instability. This protein’s absence is consistent with the described biological mechanisms of p53; the conformation of this protein depends critically on the binding of Zn^2+^ and an intrinsically fragile folding equilibrium [25], and it has also been shown that when this stabilization is not maintained, mutant p53 is glutathionylated, ubiquitinated, and subsequently targeted for proteasomal degradation [26].

Similarly, the truncating variants identified in our cohort (p.Tyr126*, p.Trp146*, p.Cys182*, p.Arg213*, p.Gly266* and the frameshift variants p.Thr140Profs*30, p.Pro151Alafs*16, p.Arg156Profs*25, and p.Asp184Alafs*62) were also observed in tumors with negative p53 immunoexpression. This pattern is expected for nonsense and frameshift variants that introduce premature termination codons (PTCs), since such transcripts typically activate the nonsense-mediated mRNA decay (NMD) pathway when the PTC is located more than 50–55 nucleotides upstream of the final exon-exon junction, thereby promoting recruitment of factors such as UPF1 and SMG1 and subsequent degradation of the mutant transcript before a stable truncated protein can accumulate [6,27]. In *TP53*, this mechanism may lead to marked transcript depletion and, consequently, to the absence of protein detectable by immunohistochemistry. Therefore, NMD could represent a biological mechanism compatible with the findings observed in the samples from our study.

However, because these alterations were detected in the heterozygous state, this finding cannot be attributed exclusively to the identified variant, since the presence of a theoretically functional remaining allele could still allow at least some degree of activity or expression. In this regard, Isermann et al. [28] indicate that *TP53* heterozygosity often progresses to subsequent loss of the wild-type allele through LOH mechanisms, an event frequently observed in tumors harboring *TP53* mutations, including CRC.

In contrast, the contact variants p.Arg248Leu, p.Arg273His/Cys, and p.Arg282Trp, as well as the conformational variant p.Tyr220Cys, showed nuclear overexpression. In CRC, this pattern is compatible with stabilization and accumulation of full-length mutant p53, a phenomenon typically associated with missense mutations and closely linked to strong nuclear staining [17,29]. For codon 273, the evidence is particularly robust in CRC. Tumors harboring Arg273 mutations, especially p.Arg273His, display a higher propensity for metastatic progression, worse survival, and a distinct oncogenic transcriptional signature [6]. In addition, recent analyses in colon tumors have linked p.Arg273His to YAP/TAZ activation, partial epithelial–mesenchymal transition (EMT) states, and a more aggressive phenotype [6,30]. Moreover, in intestinal organoids with stabilized missense mutant p53, activation of the COX-2/PGE2 pathway and transactivation of Wnt/β-catenin signaling in neighboring cells have been demonstrated, supporting the notion that mutant p53 accumulation may have functional consequences in tumor progression [31]. Although CRC-specific evidence for p.Arg248Leu remains limited, other variants affecting the same hotspot residue, particularly p.Arg248Gln and p.Arg248Trp, have been linked to GoF properties, including increased proliferation, migration, and invasion, at least in part through STAT3 hyperactivation [32,33]. In this context, the nuclear overexpression observed in p.Arg248Leu is consistent with a biologically active mutant phenotype. Regarding p.Arg282Trp, recent studies in colon tumors have included this variant among hotspot missense mutations associated with reduced survival and a more metastatic phenotype. This association may be related to the disruption of the DBD by Arg282 substitutions and their link with aggressive transcriptional states, including enrichment for CMS4-like programs in colon cancer [30].

At present, p.Tyr220Cys represents one of the *TP53* variants of greatest interest in the field of precision medicine. Structural studies have shown that substitution p.Tyr220Cys creates a surface pocket within DBD that decreases the thermal stability of the protein and promotes partial unfolding, with a consequent tendency toward self-aggregation and loss of the functional conformation of p53 [34,35]. In addition, the self-aggregated state of mutant p53 has been linked to the ability to sequester other members of the p53 family, such as p63 and p73, thereby amplifying additional oncogenic properties [36]. In our cohort, p.Tyr220Cys showed nuclear overexpression, a finding compatible with mutant protein accumulation. Importantly, this same structural pocket also constitutes a druggable vulnerability. In this context, rezatapopt (PC14586) has been developed as the first selective p53 p.Tyr220Cys reactivator, designed to bind this cavity, stabilize a wild-type-like conformation, restore DNA binding, and reactivate p53-dependent transcriptional and tumor-suppressive programs in preclinical models; moreover, the compound has shown antitumor activity and is currently under clinical evaluation in patients with advanced solid tumors harboring this variant [34,37,38].

This study has some limitations that should be considered when interpreting the results. First, although Sanger sequencing is a useful, accessible, and reproducible method for the targeted analysis of specific genomic regions, it does not provide the coverage or sensitivity of NGS-based approaches. Sanger sequencing does not reliably detect copy number alterations, such as *TP53* deletions or amplifications, or complex structural alterations. In addition, because our analysis focused on *TP53* exons 4-8, we cannot exclude the presence of additional molecular alterations in other *TP53* regions or in other genes relevant to CRC biology. Therefore, future studies using NGS and broader genomic approaches will be necessary to complement and expand these findings.

Another limitation is that p53 IHC analysis was performed in a selected subgroup of 40 tumors. This subgroup was enriched for tumors harboring oncogenic or likely oncogenic *TP53* variants. Therefore, the IHC results should not be interpreted as representative of the entire cohort or as a direct estimate of p53 immunoexpression prevalence in Mexican patients with CRC. This selection strategy also limits the possibility of performing robust statistical associations between p53 expression and *TP53* mutational status, since these correlations could be biased.

Finally, survival analyses, prognostic evaluation, and therapeutic response data were not included; therefore, the clinical implications of these alterations in the present cohort should be interpreted with caution. Future studies including larger cohorts, broader genomic approaches, and clinical follow-up will be necessary to more accurately determine the prognostic or therapeutic relevance of these findings. Despite these limitations, our results provide relevant local evidence on the spectrum of *TP53* alterations in Mexican patients with CRC and support the value of integrating molecular characterization with immunohistochemical assessment for a better biological interpretation of these tumors.

## 5. Conclusions

In conclusion, in this Mexican cohort of patients with sporadic CRC, sequencing of *TP53* exons 4–8 identified heterozygous variants in 75% of patients (106/142), including 31 cases harboring oncogenic variants mainly located within the DNA-binding domain (DBD) and involving hotspot residues such as Arg175, Tyr220, Gly245, Arg248, Arg273, and Arg282. p53 immunohistochemistry, performed in a selected subgroup, showed nuclear positivity in 23% of tumors (9/40), whereas most cases showed no detectable expression.

These findings provide local evidence on the molecular- and protein-level heterogeneity of *TP53* in Mexican patients with colorectal cancer and support the value of integrating targeted sequencing with immunohistochemical assessment for the biological interpretation of these tumors.

## Figures and Tables

**Table 1:** Demographic and clinicopathological characteristics of 142 colorectal cancer patients.

Characteristic	Value
N	142
Age, mean ± SD (years)	58.9 ± 16.2
Age range (years)	15–96
Age group	
<50 years	34 (24%)
≥50 years	100 (70%)
Missing data	8 (6%)
Sex	
Male	76 (54%)
Female	63 (44%)
Missing data	3 (2%)
Tumor location	
Rectum	55 (39%)
Colon	77 (54%)
Missing data	10 (7%)
Differentiation grade	
Well differentiated	3 (2%)
Moderately differentiated	105 (74%)
Poorly differentiated	18 (13%)
Missing data	16 (11%)
TNM stage	
0–II	45 (32%)
III–IV	82 (58%)
Missing data	15 (10%)

SD, standard deviation; TNM, tumor–node–metastasis. Clinical classification and TNM stage were determined according to AJCC criteria, 9th edition.

## Data Availability

The data presented in this study are available on request from the corresponding author due to privacy and ethical restrictions.

## Supplementary Materials

The following supporting information can be downloaded at: https://www.mdpi.com/article/10.3390/cancers18111678/s1, Table S1. TP53 variants identified in exons 4–8 and flanking intronic regions. Table S2. TP53 variants according to p53 immunohistochemical expression status.

## Abbreviations

The following abbreviations are used in this manuscript:

CRC	Colorectal cancer
CIN	Chromosomal instability
*TP53*	Tumor protein p53 gene
p53	Tumor protein p53
DBD	DNA-binding domain
IHC	Immunohistochemistry
IRS	Immunoreactivity Score
PCR	Polymerase chain reaction
HGVS	Human Genome Variation Society
MANE	Matched Annotation from NCBI and EMBL-EBI
GoF	Gain-of-function
PTC	Premature termination codon
NMD	Nonsense-mediated mRNA decay
LOH	Loss of heterozygosity
EMT	Epithelial–mesenchymal transition
COX-2	Cyclooxygenase-2
PGE2	Prostaglandin E2
TNM	Tumor-node-metastasis
FDA	Food and Drug Administration
TAD2	Transactivation domain 2
rsID	Reference SNP cluster identifier
SECIHTI	Secretaría de Ciencia, Humanidades, Tecnología e Innovación
