# Supplementary material for: Molecular Characterization of TP53 Variants in Exons 4-8 and p53 Immunoexpression in a Mexican Colorectal Cancer Cohort
Source: Cancers (Basel). 2026 May 22;18(11):1678. doi: 10.3390/cancers18111678 (PMC13255983; doi:10.3390/cancers18111678)
Supplement: Supplementary file 1 [file cancers-18-01678-s001.zip › cancers-4310771-supplementary.pdf]

Table S1. *TP53* variants identified in exons 4-8 and flanking intronic regions

rsID	Region	Domain	HGVS c.	HGVS p.	Molecular Consequence	Horak et al. [14] classification	OncoKB Functional annotation
rs1478438654	Intron 4	-	c.376-27A>C	p.?	Intronic	Likely benign	-
rs2909430	Intron 4	-	c.376-91G>C	p.?	Intronic	Likely benign	-
rs59758982	Intron 3	-	c.96+41_97-54del	p.?	Intronic	Benign	-
rs17883323	Intron 3	-	c.97-29C>A	p.?	Intronic	Benign	-
rs12947788	Intron 7	-	c.782+72C>T	p.?	Intronic	Benign	-
rs1800370	Exon 4	TAD2	c.108G>A	p.Pro36=	Synonymous	Benign	-
rs1555526742	Exon 4	TAD2	c.160T>C	p.Phe54Leu	Missense	Likely benign	-
rs775185978	Exon 4	-	c.207T>C	p.Ala69=	Synonymous	Likely benign	-
rs786203513	Exon 4	-	c.210T>C	p.Ala70=	Synonymous	Likely benign	-
rs1555526701	Exon 4	-	c.213C>T	p.Pro71=	Synonymous	Likely benign	-
rs1042522	Exon 4	-	c.215C>G	p.Pro72Arg	Missense	Benign	-
rs372397095	Exon 4	-	c.246G>T	p.Pro82=	Synonymous	Likely benign	-
rs751978853	Exon 4	-	c.354A>T	p.Thr118=	Synonymous	Likely benign	-
rs1567554500	Exon 5	DBD	c.378C>G	p.Tyr126*	Nonsense	Oncogenic	Loss-of-function Level Px1
rs730881999	Exon 5	DBD	c.380C>T	p.Ser127Phe	Missense	Oncogenic	Loss-of-function Level Px1
rs879254214	Exon 5	DBD	c.393_395del	p.Asn131del	Inframe deletion	Oncogenic	Loss-of-function Level Px1
rs747342068	Exon 5	DBD	c.394A>T	p.Lys132*	Nonsense	Oncogenic	Likely Loss-of-function Level Px1
rs780442292	Exon 5	DBD	c.401T>C	p.Phe134Ser	Missense	Likely oncogenic	Likely Loss-of-function Level Px1
rs587781991	Exon 5	DBD	c.404G>T	p.Cys135Phe	Missense	Oncogenic	Likely Loss-of-function Level Px1
rs1567557016	Exon 5	DBD	c.417del	p.Thr140Profs*30	Frameshift deletion	Oncogenic	Likely Loss-of-function Level Px1
rs1206165503	Exon 5	DBD	c.437G>A	p.Trp146*	Nonsense	Oncogenic	Likely Loss-of-function Level Px1
rs137852791	Exon 5	DBD	c.450_459del	p.Pro151Alafs*16	Frameshift deletion	Oncogenic	Likely Loss-of-function Level Px1
rs786202752	Exon 5	DBD	c.464C>A	p.Thr155Asn	Missense	Likely oncogenic	Likely Loss-of-function Level Px1
-	Exon 5	DBD	c.466dup	p.Arg156Profs*25	Frameshift duplication	Oncogenic	Likely Loss-of-function Level Px1
rs28934578	Exon 5	DBD	c.524G>T	p.Arg175Leu	Missense	Oncogenic	Loss-of-function Level Px1
rs1064796257	Exon 5	DBD	c.546C>A	p.Cys182*	Nonsense	Oncogenic	Likely Loss-of-function Level Px1
rs1597369519	Exon 5	DBD	c.551_554del	p.Asp184Alafs*62	Frameshift deletion	Oncogenic	Likely Loss-of-function Level Px1
rs876660254	Exon 6	DBD	c.568C>A	p.Pro190Thr	Missense	Likely oncogenic	Likely Loss-of-function Level Px1
rs1464727668	Exon 6	DBD	c.623A>T	p.Asp208Val	Missense	Likely oncogenic	Likely Loss-of-function Level Px1
rs397516436	Exon 6	DBD	c.637C>T	p.Arg213*	Nonsense	Oncogenic	Likely Loss-of-function Level Px1
rs1800372	Exon 6	DBD	c.639A>G	p.Arg213=	Synonymous	Benign	-
rs587781386	Exon 6	DBD	c.642T>G	p.His214Gln	Missense	Likely oncogenic	Likely Gain-of-function
rs587782177	Exon 6	DBD	c.644G>A	p.Ser215Asn	Missense	Oncogenic	Likely Loss-of-function Level Px1
rs121912666	Exon 6	DBD	c.659A>G	p.Tyr220Cys	Missense	Oncogenic	Loss-of-function Level 3A | Level Px1 | FDA Level 3
rs730882026	Exon 7	DBD	c.707A>G	p.Tyr236Cys	Missense	Oncogenic	Likely Loss-of-function Level Px1

rs28934575	Exon 7	DBD	c.733G>A	p.Gly245Ser	Missense	Oncogenic	Loss-of-function Level Px1
rs28934575	Exon 7	DBD	c.733G>T	p.Gly245Cys	Missense	Oncogenic	Inconclusive
rs11540652	Exon 7	DBD	c.743G>T	p.Arg248Leu	Missense	Oncogenic	Likely Loss-of-function Level Px1
rs1057519990	Exon 8	DBD	c.796G>T	p.Gly266*	Nonsense	Oncogenic	Likely Loss-of-function Level Px1
rs121913343	Exon 8	DBD	c.817C>T	p.Arg273Cys	Missense	Oncogenic	Likely Loss-of-function Level Px2
rs28934576	Exon 8	DBD	c.818G>A	p.Arg273His	Missense	Oncogenic	Loss-of-function Level Px1
rs28934574	Exon 8	DBD	c.844C>T	p.Arg282Trp	Missense	Oncogenic	Likely Loss-of-function Level Px1
rs786201059	Exon 8	DBD	c.856G>A	p.Glu286Lys	Missense	Oncogenic	Likely Loss-of-function Level Px1

rsID, Reference SNP cluster ID, HGVS, Human Genome Variation Society. c., coding DNA sequence. p., protein sequence. p.?, protein consequence not predicted. DBD, DNA-binding domain; TAD2, transactivation domain 2. functional annotations correspond to the evidence labels reported in the original dataset (OncoKB when available [12,13]).

Table S2. *TP53 variants according to p53 immunohistochemical expression status*

p53 IHC status	rs ID	HGVS	Horak et al. [14] classification	IRS[1]
Expression	rs730881999	NM_000546.6(TP53):c.380C>T (p.Ser127Phe)	Likely oncogenic	6
Expression	rs747342068	NM_000546.6(TP53):c.394A>T (p.Lys132*)	Oncogenic	12
Expression	rs876660254	NM_000546.6(TP53):c.568C>A (p.Pro190Thr)	Likely oncogenic	12
Expression	rs121912666	NM_000546.6(TP53):c.659A>G (p.Tyr220Cys)	Oncogenic	12
Expression	rs11540652	NM_000546.6(TP53):c.743G>T (p.Arg248Leu)	Oncogenic	12
Expression	rs28934576	NM_000546.6(TP53):c.818G>A (p.Arg273His)	Oncogenic	9
Expression	rs28934574	NM_000546.6(TP53):c.844C>T (p.Arg282Trp)	Oncogenic	9
No expression	rs17883323	NC_000017.11(NM_000546.6):c.97-29C>A p.?	Benign	-
No expression	rs2909430	NC_000017.11(NM_000546.6):c.376-91G>C p.?	Likely benign	-
No expression	rs1555526742	NM_000546.6(TP53):c.160T>C (p.Phe54Leu)	Likely benign	-
No expression	rs775185978	NM_000546.6(TP53):c.207T>C (p.Ala69=)	Likely benign	-
No expression	rs1042522	NM_000546.6(TP53):c.215C>G (p.Pro72Arg)	Benign	-
No expression	rs1567554500	NM_000546.6(TP53):c.378C>G p.(Tyr126*)	Oncogenic	-
No expression	rs879254214	NM_000546.6(TP53):c.393_395del (p.Asn131del)	Oncogenic	-
No expression	rs780442292	NM_000546.6(TP53):c.401T>C (p.Phe134Ser)	Likely oncogenic	-
No expression	rs587781991	NM_000546.6(TP53):c.404G>T (p.Cys135Phe)	Oncogenic	-
No expression	rs1567557016	NM_000546.6(TP53):c.417del (p.Thr140Profs*30)	Oncogenic	-
No expression	rs1206165503	NM_000546.6(TP53):c.437G>A (p.Trp146*)	Oncogenic	-
No expression	rs137852791	NM_000546.6(TP53):c.450_459del (p.Pro151Alafs*16)	Oncogenic	-
No expression	rs786202752	NM_000546.6(TP53):c.464C>A (p.Thr155Asn)	Likely oncogenic	-
No expression	-	NM_000546.6(TP53):c.466dup (p.Arg156Profs*25)	Oncogenic	-
No expression	rs28934578	NM_000546.6(TP53):c.524G>T (p.Arg175Leu)	Oncogenic	-
No expression	rs1064796257	NM_000546.6(TP53):c.546C>A (p.Cys182*)	Oncogenic	-
No expression	rs1597369519	NM_000546.6(TP53):c.551_554del (p.Asp184Alafs*62)	Oncogenic	-
No expression	rs397516436	NM_000546.6(TP53):c.637C>T (p.Arg213*)	Oncogenic	-
No expression	rs587781386	NM_000546.6(TP53):c.642T>G (p.His214Gln)	Likely oncogenic	-
No expression	rs587782177	NM_000546.6(TP53):c.644G>A (p.Ser215Asn)	Oncogenic	-
No expression	rs730882026	NM_000546.6(TP53):c.707A>G (p.Tyr236Cys)	Oncogenic	-
No expression	rs28934575	NM_000546.6(TP53):c.733G>A (p.Gly245Ser)	Oncogenic	-
No expression	rs121913343	NM_000546.6(TP53):c.817C>T (p.Arg273Cys)	Oncogenic	-

[1] Two p53-positive tumor samples showed no *TP53* variants in the analyzed regions. HGVS, Human Genome Variation Society. IRS, Immunoreactive Score. IHC, Immunohistochemistry.
